# Postpartum anxiety, depression and social health: findings from a population-based survey of Australian women

**DOI:** 10.1186/1471-2458-10-771

**Published:** 2010-12-20

**Authors:** Jane Yelland, Georgina Sutherland, Stephanie J Brown

**Affiliations:** 1 Healthy Mothers Healthy Families research group, Murdoch Childrens Research Institute, Flemington Road, Parkville Victoria, 3052, Australia; 2 Department of General Practice and School of Population Health, University of Melbourne, Parkville Victoria, 3052, Australia

## Abstract

**Background:**

Whilst the prevalence and correlates of postpartum depression are well established, far less is known about postpartum anxiety. Studies have described the association between socio-demographic factors and postpartum depression, yet few have explored the association between stressors in women's lives around the time of having a baby and maternal psychological morbidity. This study aimed to describe the population prevalence of postpartum depression, anxiety, co-morbid anxiety and depression and social health issues; and to examine the association between postpartum psychological and social health issues experienced in the six months following birth.

**Methods:**

Population-based survey of all women who gave birth in Victoria and South Australia in September/October 2007. Women were mailed the survey questionnaire six months following birth. Anxiety and depression were measured using the Depression Anxiety Stress Scales (DASS-21).

**Results:**

Questionnaires were completed by 4,366 women. At six months postpartum the proportion of women scoring above the 'normal' range on the DASS-21 was 12.7% for anxiety,17.4% for depression, and 8.1% for co-morbid depression and anxiety. Nearly half the sample reported experiencing stressful life events or social health issues in the six months following birth, with 38.3% reporting one to two and 8.8% reporting three or more social health issues. Women reporting three or more social health issues were significantly more likely to experience postnatal anxiety (Adj OR = 4.12, 95% CI 3.0-5.5) or depression (Adj OR = 5.11, 95% CI = 3.9-6.7) and co-morbid anxiety *and *depression (Adj OR = 5.41, 95% CI 3.8-7.6) than women who did not report social health issues.

**Conclusions:**

Health care providers including midwives, nurses, medical practitioners and community health workers need to be alert to women's social circumstances and life events experienced in the perinatal period and the interplay between social and emotional health. Usual management for postpartum mental health issues including Cognitive Behavioural Therapy and pharmacological approaches may not be effective if social health issues are not addressed. Coordinated and integrated perinatal care that is responsive to women's social health may lead to improvements in women's emotional wellbeing following birth.

## Background

Having a baby is a significant transitional life event, especially for women having their first child. It involves changes in relationships between couples and within families, and is commonly a cause of additional financial stress, even among households with relatively high incomes [1]. The impact of stressful life events and social health issues on maternal psychological morbidity, such as depression, has been identified in several studies. O'Hara [2] noted that stressful life events experienced in pregnancy or early postpartum was a strong predictor of postnatal depressive symptoms. A Swedish study identified that one of the factors associated with depressive symptoms in a large national sample was two or more stressful life events in the year prior to pregnancy [3]. Studies from Australia [4] and the USA [5] have also found associations between experiencing stressful life events and maternal depression.

Internationally there is increasing recognition that maternal depression is a major public health issue, with potential long term consequences for women's health and the health of her infant and other family members [6,7]. Concern about under-detection and poor management of maternal depression has led to development of guidelines for assessment and screening of pregnant and postnatal women in many Western countries. Other psychosocial morbidity, including anxiety, has generally been overlooked in these national responses. This is despite evidence that symptoms of depression and anxiety commonly co-occur [8,9] and that this co morbidity may be an indicator of severity of psychological ill-health [10]. Antenatal anxiety has been shown to be associated with preterm birth [11] and predict postnatal depressive disorders [8]. Studies examining outcomes associated with maternal postnatal anxiety suggest exposure is related to adverse psychological problems in children [12].

Previous research have used socio-demographic determinants (i.e. income, educational attainment) to describe social disparities in maternal emotional wellbeing [13,14]. The few studies examining perinatal stressful life events and psychological morbidity have focussed on maternal depression alone [2-5,15,16]. This study addresses limitations of previous research, drawing on data from a large Australian population based survey of recent mothers to examine anxiety, depression and co-morbid symptoms at six months postpartum, together with women's experiences of stressful life events and social health issues. The primary aims of analyses presented in the paper are to: (i) determine the prevalence of maternal anxiety and depressive symptoms, and co-morbid symptoms, at six months postpartum; (ii) investigate the association between postpartum anxiety and depression and stressful life events and social health issues experienced in the six months following birth.

## Method

### Sample

The Healthy Mothers Healthy Families Survey questionnaire was mailed to all women who gave birth in a four week period in Victoria and an eight week period in South Australia in September/October 2007, excluding those who had a stillbirth, or whose baby was known to have died. All public and private maternity hospitals and homebirth practitioners were asked to facilitate the study by distributing the questionnaires to women who gave birth under their care in the study period. All hospitals with births in the study period (n = 110 hospitals) agreed to participate, however one hospital withdrew from the study at the time of the mail-out. Questionnaires, together with a covering letter inviting women to take part, an explanation of the study in six community languages (Arabic, Vietnamese, Cantonese, Mandarin, Somali and Turkish) and a reply paid envelope for returning the questionnaire free of charge, were posted to women at around 6 months postpartum. Two reminders were sent at 2-week intervals; the second of these included a repeat copy of the questionnaire. The survey questionnaire was in English only.

Research ethics approval was obtained from the ethics committee of the Victorian Department of Human Services, the South Australian Department of Health, the University of South Australia, the Royal Children's Hospital and ten participating hospitals.

### Questionnaire

The questionnaire covered women's views of antenatal, intrapartum and postnatal care. Data were also collected on reproductive history, maternal socio-demographic characteristics, and maternal psychosocial health issues in pregnancy and postnatally. Maternal socio-demographic characteristics included: maternal country of birth, Aboriginal or Torres Strait Islander, completion of secondary school, household income, health care concession card holder, relationship status and health insurance status. The reported estimate of before tax household income has been adjusted by an equivalence factor (using the modified OECD equivalence scale) [17] to account for households of different size (number of people) and composition (adults and children). The questionnaire was developed drawing on questionnaires used in three previous Victorian Surveys of Recent Mothers [18-20] and standardised instruments as outlined below. Piloting was undertaken to assess acceptability with diverse groups of recent mothers.

### Measurement of postnatal anxiety and depression using the DASS-21

The Depression Anxiety Stress Scales (DASS) [21] is a self-report instrument designed to measure the negative emotional states of depression, anxiety and stress. The DASS depression scale has been shown to correlate strongly with the Beck Depression Inventory-(BDI-II, r = .74), and the DASS anxiety scale with the Beck Anxiety Inventory (r = .81) [21,22].

The 21 item form of the DASS was incorporated into the questionnaire to measure severity of symptoms common to anxiety and depression and the prevalence of co-morbid depression and anxiety. Women were asked to use a 4-point severity/frequency scale to rate the extent to which they had experienced each symptom over the past week from 'never' to 'most of the time'.

### Measurement of stressful life events and social health issues

The questionnaire included a list of stressful life events and social health issues drawing on items from the Pregnancy Risk Assessment and Monitoring Study (PRAMS) [23] and a review of the impact of life events and social health issues on health in the perinatal period. Women were asked "Have any of the following things happened to you in the months since your baby was born?' and asked to tick yes or no to the list of items including major life events, such as separation and divorce, moving house, losing your job, death of a close family member or friend and social health issues, such as having a lot of bills you couldn't pay, not having enough money to buy food, legal troubles or being involved in a court case, serious family conflict or being homeless.

### Analysis

To assess the representativeness of the sample we compared data on the social characteristics of study participants with routinely collected data from the Victorian Perinatal Data Collection Unit and South Australian Pregnancy Outcome Unit for all women who gave birth in the study period, excluding women who had a stillbirth or neonatal death.

Data were analysed using STATA version 11.0 [24] and involved the calculation of unadjusted and adjusted odds ratios and 95% confidence intervals. Level of statistical significance was p < 0.01. Scores for the DASS-21 sub-scales of depression and anxiety were derived by totalling the scores for each sub-scale and multiplying by two to ensure consistent interpretation with the longer 42 item version [21]. We classified women according to the recommended scoring system using cut-off values to classify participants into the following categories: normal (0-9 for depression and 0-7 for anxiety), mild (10-13 for depression and 8-9 for anxiety), moderate (14-20 for depression and 10-14 for anxiety), severe (21-27 for depression and 15-19 for anxiety), and extremely severe (≥28 for depression and ≥20 for anxiety). We have referred to women within the 'normal' range on the DASS-21 for depressive or anxiety symptoms as *non-depressed *or *non-anxious *respectively. Women, who scored in the 'mild' to 'extremely severe' ranges, were referred to as *depressed *or *anxious*. Analysis is based on this dichotomy (i.e. 'normal range' versus 'mild to extremely severe' symptoms).

Multivariable logistic regression was used to assess the relationship between number of stressful life events and social health issues as the exposure of main interest, and depression, anxiety, and co-morbid depression and anxiety as the primary outcome variables. Three separate models were developed adjusting for maternal socio-demographic characteristics. Women with missing responses on any variable were excluded from these analyses.

## Results

### Response & characteristics of the sample

**Figure 1:**
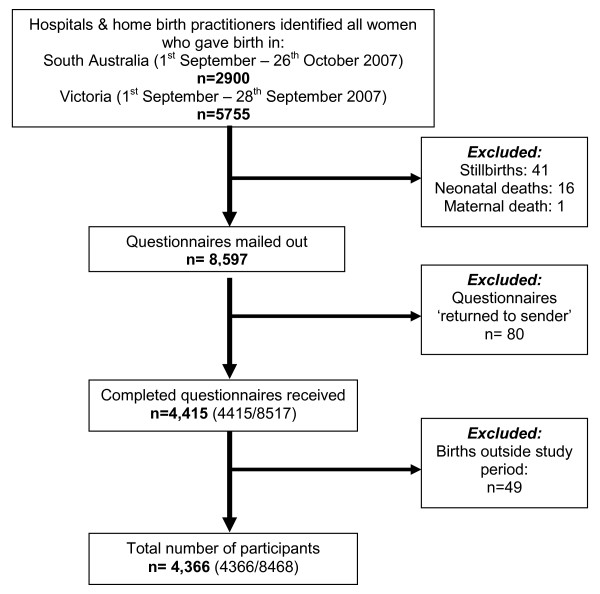
**Flow chart of survey participation**.

Questionnaires were mailed to 8,597 women six month after childbirth. The adjusted response fraction excluding questionnaires that had been 'returned to sender', duplicate responses and women who gave birth outside the study period was 52% (4366/8468). See Figure 1.

Table 1 shows selected demographic and obstetric characteristics of the sample. Participants were aged between 16 and 46 years, with a mean age of 31 years. More than half the sample was multiparous (56%). One in five participants (19%) were born outside Australia, one in ten (12%) in countries where English is not the primary language. Three-quarters of participants (76%) were married and 19% were living with a partner. Forty-eight percent of women had private health insurance, with the remainder covered by Medicare, Australia's universal health insurance scheme.

**Table 1:** Social and reproductive characteristics of participants in the HMHF Survey (n = 4,366)

	**No**.	%
**Maternal age (years)**		
16-24	416	9.9
25-29	1053	25.0
30-34	1604	38.1
≥35	1138	27.0

**Relationship status**		
Married	3318	76.3
Living with partner	835	19.2
Not living with partner/divorced/separated/widowed/single	198	4.5

**Indigenous status**		
Non-Aboriginal	4051	99.2
Aboriginal and/or Torres Strait Islander	31	0.8

**Country of birth**		
Australia	3521	81.5
Overseas - English speaking	272	6.3
Overseas - Non English speaking background	526	12.2

**Education**		
Completed secondary school	3429	79.3
Did not complete secondary school	894	20.7

Equivalised Household Income*		
< $20,000	938	23.9
$20,000-$40,000	1958	50.6
> $40,000	979	25.5

**Health Care Card**		
No	3597	83.1
Yes	734	16.9

**Private health insurance for birth**		
No	2250	51.8
Yes	2092	48.2

**Parity**		
Multiparous	2425	55.7
Primiparous	1941	44.3

**Method of birth**		
Spontaneous vaginal birth	2338	53.7
Instrumental vaginal birth	602	13.8
Caesarean section, no labour	801	18.4
Caesarean section, in labour	615	14.1

**Birthweight of the infant**		
< 2500g	179	4.3
2500-3499g	2023	48.7
3500-3999g	1390	33.5
≥4000	560	13.5

**Plurality**		
Singleton	4275	98.4
Twin	64	1.5
Triplet	1	< 0.1

Denominators vary because of missing values

*Adjusted by family size and composition using OECD modified equivalence scale

Women taking part in the survey were largely representative in terms of parity, method of birth and infant birthweight compared with women who gave birth in the study period according to data collected by the Perinatal Data Collection Unit in Victoria and the Pregnancy Outcome Unit in South Australia. Women born overseas of non-English speaking background, Aboriginal and Torres Strait Islander women, women under the age of 25 years and single women were under-represented. The estimated response fractions for women born overseas of non-English speaking background, and young women (under 25 years) were 34% and 30% respectively.

### Depression and anxiety

**Figure 2:**
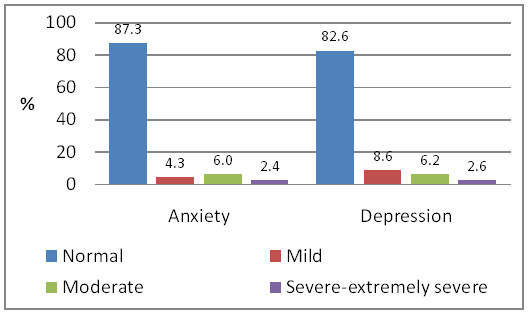
**Prevalence of anxiety and depression at six months postpartum (n = 4,269)**.

At six months postpartum the proportion of women reporting symptoms in the 'mild' to 'extremely severe' range on the DASS-21 scales was 12.7% for anxiety and 17.4% for depression, 8.1% of women reported co-morbid symptoms, i.e. combined anxiety and depressive symptoms. Of the 918 women who experienced symptoms of anxiety and/or depression, 385/918 (41.9%) scored as depressed alone, 189/918 (20.6%) scored as anxious alone, and 344/918 (37.5%) scored as both depressed and anxious. Around 2% of women could not be classified due to missing values. Figure 2 shows the severity of anxiety and depressive symptoms, according to scores on the DASS 21.

### Stressful life events and social health issues

Nearly half of women reported experiencing one or more stressful life events or social health issues in the six months following birth (47.6%, 2076/4359), 37.8% (1646/4359) reported one or two stressful life events or social health issues and 9.9% (430/4359) reported three or more social health issues. Data were missing for seven women who did not complete this section of the questionnaire. Reported life events and social health issues are shown in Table 2. The most common life events, affecting one in ten women, were death or illness of friend or family member, and moving to a new house/place to live. Common social health issues affecting around one in ten women were: having a lot of bills you couldn't pay, and serious conflict between members of your family. Nearly five percent of women reported not having enough money to buy food.

**Table 2:** Number and proportion of women reporting stressful life events and social health issues in the six months following birth (n = 4,356)

Social health issue	Number	%
***Emotional stressors***		

Major illness or injury of close family member or friend	645	14.8

Death of a close family member/friend	440	10.1

You had a major illness or injury	216	4.9

***Relationship stressors***		

There was serious conflict between members of your family	432	9.9

You started a new close personal relationship, moved in with your partner or got married	110	2.5

You or your partner had legal troubles and were involved in a court case	111	2.5

You or your partner had trouble with alcohol or illicit drugs	94	2.2

Separation or divorce	64	1.5

Your partner said he/she did not want your baby	46	1.1

You or your partner had trouble with gambling	27	0.6

***Traumatic stressors***		

You were humiliated or emotional abused in other ways by your partner or ex-partner	149	3.4

You were kicked, hit, slapped or otherwise physically hurt by your partner or ex-partner	55	1.3

You were forced to take part in unwanted sex	16	0.4

Someone else (other than your partner or ex-partner) pushed, grabbed, shoved, kicked or hit you	15	0.3

You were homeless	7	0.2

***Financial stressors***		

You had a lot of bill's that I couldn't pay	522	12.0

You moved to a new house/new place to live	419	9.6

You were unable to return to work as planned	225	5.2

You didn't have enough money for food	192	4.4

Your partner loss his or her job	137	3.2

Table 3 describes the relationship between the number of stressful life events and social health issues, and social and obstetric characteristics. Women who reported three or more social health issues were more likely to: be under 25 years (OR 2.63 [95% CI 2.1-3.3]), unmarried (OR 1.97 [95% CI 1.7-2.3]), of Aboriginal or Torres Strait Islander origin (OR 2.69 [95% CI 1.2-5.9]), not to have completed secondary school (OR 1.43 [95% 1.2-1.6]), to have a low income (OR 1.94 [95% CI 1.6-2.3]), health care concession card (OR 2.14 [95% CI 1.8-2.5]) and not to have private health insurance (OR 1.48 [95% CI 1.3-1.7]).

**Table 3:** Relationship between number of stressful life events and social health issues, and social and obstetric characteristics

	No social health issues (n = 2238)		One - two social health issues (n = 1646)		Three or more social health issues (n = 430)	
	**No**.	**%**	**No**.	**%**	**No**.	**%**

**Maternal age (yrs)**						
16-24	140	33.7	178	42.9	97	23.4
25-29	528	50.2	410	39.0	113	10.7
30-34	917	57.3	579	36.2	105	6.6
≥35	621	54.6	423	37.2	93	8.2

**Relationship status**						
Married	1868	56.3	1208	36.4	240	7.2
Living with partner	353	42.3	359	42.9	123	14.7
Not living with partner/single/widowed/divorced/separated	54	27.5	76	38.8	66	33.7

**Country of birth**						
Australia	1807	51.4	1346	38.3	365	10.4
Overseas - English speaking	154	56.8	102	37.6	15	5.5
Overseas - NESB	302	57.6	175	33.4	47	8.9

**Indigenous status**						
Aboriginal or Torres Strait Islander	9	29.0	12	3.7	10	32.3
Not Aboriginal or Torres Strait Islander	2120	52.4	1530	37.8	395	9.8

**Education**						
Completed secondary school	1858	54.3	1271	37.1	295	8.6
Did not complete secondary school	405	45.3	357	39.9	131	14.7

Equivalised household income*						
< $20,000	366	39.0	386	41.1	186	19.8
$20,000-$40,000	1084	55.4	721	36.8	152	7.8
> $41,000	578	59.2	355	36.3	44	4.5

**Health care card**						
No	1919	56.6	1251	36.9	221	6.5
Yes	358	37.8	382	40.3	207	21.9

**Private health insurance for birth**						
Yes	1203	57.5	767	36.7	121	5.8
No	1073	37.8	866	38.6	307	13.7

**Parity**						
Multiparous	1294	53.4	890	36.7	238	9.8
Primiparous	989	51.1	756	39.0	192	9.9

*Adjusted by family size and composition using OECD modified equivalence scale

### Association between depression, anxiety and social health

Associations between maternal socio-demographic characteristics, stressful life events and social health issues, and anxiety and depression are shown in Table 4. Unadjusted odds ratios indicate that women who scored above the cut-off score for depressive symptoms were more likely to: be young (under 25 years of age), living with their partner or single, divorced or separated rather than married, not to have completed secondary school and to have a health care concession card. Women having their first baby were less likely to have depressive symptoms than women having a second or subsequent baby. There was a very marked association between number of stressful life events and social health issues and depressive symptoms. Reporting three or more stressful life events or social health issues was associated with greater than a five-fold increase in odds of depressive symptoms. Odds were also significantly raised for women reporting one or two stressful life events or social health issues.

**Table 4:** Associations of depression (n = 4063) and anxiety (n = 4078) symptoms with social characteristics, parity and social health issues at six months postpartum

	*Depressed* *DASS ≥10* (n = 692)		OR (95% CI) of depression	Adjusted OR (95% CI)	*Anxious* *DASS ≥8* (n = 505)		OR (95% CI) of anxiety	Adjusted OR (95% CI)
	**No**.	%			**No**.	%		
**Stressful life events & social** **health issues experienced in 6** **months postnatally**								
None	241	11.3	1.00 Ref	1.00 Ref	170	7.9	1.00 Ref	1.00 Ref
One-two	282	18.5	**1.78 (1.5-2.1)**	**1.75 (1.4-2.1)**	214	13.9	**1.88 (1.5-2.3)**	**1.82 (1.5-2.3)**
Three or more	169	43.0	**5.95 (4.7-7.6)**	**5.37 (4.1-6.9)**	121	30.6	**5.14 (3.9-6.7)**	**4.27 (3.2-5.6)**

**Maternal age (yrs)**								
16-24	97	25.0	**1.70 (1.3-2.2)**	1.14 (0.8-1.6)	77	20.0	**2.01 (1.5-2.7**)	1.15 (0.8-1.6)
25-29	173	17.1	1.05 (0.8-1.3)	0.97 (0.8-1.2)	134	13.1	1.22 (0.9-1.5)	1.03 (0.8-1.4)
30-34	254	16.4	1.00 Ref	1.00 Ref	172	11.0	1.00 Ref	1.00 Ref
≥35	168	15.1	0.91 (0.7-1.1)	0.82 (0.7-1.1)	122	10.9	0.99 (0.8-1.3)	0.90 (0.7-1.2)

**Relationship status**								
Married	480	15.4	1.00 Ref	1.00 Ref	350	11.2	1.00 Ref	1.00 Ref
Living with partner	163	21.2	**1.47 (1.2-1.8)**	1.24 (0.9 -1.5)	118	15.2	**1.42 (1.1-1.8)**	1.09 (0.8-1.4)
Not living with partner/single/	49	27.8	**2.12 (1.5-2.9)**	1.17 (0.8-1.7)	37	21.1	**2.12 (1.4-3.1)**	0.99 (0.6-1.6)
widowed/divorced/separated								

**Country of birth**								
Australia	563	16.8	1.00 Ref	1.00 Ref	403	11.9	1.00 Ref	1.00 Ref
Overseas - English speaking	42	16.2	0.95 (0.7-1.3)	1.12 (0.8-1.6)	20	7.7	0.61 (0.4-1.0)	0.69 (0.4-1.1)
Overseas - NESB	87	19.3	1.18 (0.9-1.5)	**1.34 (1.0-1.8)**	82	18.3	**1.65 (1.3-2.1)**	**1.84 (1.4-2.4**)

**Education**								
Completed secondary school	524	16.2	1.00 Ref	1.00 Ref	361	11.1	1.00 Ref	1.00 Ref
Did not complete secondary school	168	20.5	**1.33 (1.1-1.6)**	1.05 (0.8-1.3)	144	17.5	**1.70 (1.4-2.1)**	1.36 (0.9-1.6**)**

**Health care card**								
No	487	15.2	1.00 Ref	1.00 Ref	328	10.2	1.00 Ref	1.00 Ref
Yes	205	23.9	**1.74 (1.5-2.1)**	1.15 (0.9-1.4)	177	20.5	**2.26 (1.8-2.8)**	**1.51 (1.2-1.9)**

**Parity**								
Multiparous	416	18.4	1.00 Ref	1.00 Ref	289	12.7	1.00 Ref	1.00 Ref
Primiparous	276	15.3	**0.80 (0.7-0.9**)	**0.72 (0.6 -0.9**)	216	11.9	0.92 (0.8-1.1)	0.86 (0.7-1.1)

A similar pattern is apparent for women with symptoms of anxiety. There was a two-fold increase in the unadjusted odds of reporting anxiety symptoms in women reporting one to two stressful life events or social health issues, rising to a five fold increase for women reporting three or more issues or events. Women born overseas in a country where English is not the primary community language were more likely to report anxiety symptoms than women born in Australia. There was no association between anxiety symptoms and parity (Table 4).

When compared with women with no psychological morbidity, symptoms of co-morbid anxiety *and *depression were associated with young maternal age, not being married, not having completed secondary school, having a health care concession card and experiencing one or more social health issues following birth (Table 5).

**Table 5:** Factors associated with co-morbid anxiety and depression at six months postpartum (n = 4052)

	No psychological morbidity No. (%)	Co-morbid anxiety & depression No. (%)	Unadjusted OR (95% CI)	Adjusted OR (95% CI)
**Stressful life events & social health issues** **experienced in 6 months postnatally**				
None	2040 (95.5)	95 (4.5)	1.00 Ref	1.00 Ref
One-two	1395 (91.6)	128 (8.4)	**1.97 (1.5-2.6)**	**1.88 (1.4-2.5)**
Three or more	298 (75.5)	96 (24.4)	**6.92 (5.1-9.4)**	**5.77 (4.1-8.0)**

**Maternal age (yrs)**				
16-24	331 (85.9)	54 (14.1)	**2.09 (1.5-2.9)**	1.15 (0.8-1.7)
25-29	931 (92.0)	81 (8.0)	1.11 (0.8-1.5)	0.77 (0.7-1.3)
30-34	1435 (92.7)	112 (7.3)	1.00 Ref	1.00 Ref
≥35	1036 (93.5)	72 (6.5)	0.89 (0.6-1.2)	0.78 (0.6-1.1)

**Relationship status**				
Married	2984 (93.1)	214 (6.9)	1.00 Ref	1.00 Ref
Living with partner	688 (89.7)	79 (10.3)	**1.55 (1.2-2.1)**	1.14 (0.8-1.6)
Not living with partner/single/widowed/divorced/separated	151 (85.3)	26 (14.7)	**2.32 (1.5-3.6)**	0.96 (0.6-1.7)

**Country of birth**				
Australia	3090 (92.3)	257 (7.7)	1.00 Ref	1.00 Ref
Overseas - English speaking	242 (93.8)	16 (6.2)	0.79 (0.5-1.3)	0.97 (0.6-1.6)
Overseas - NESB	401 (89.7)	46 (10.3)	1.37 (0.9-1.9)	**1.56 (1.1-2.2)**

**Education**				
Completed secondary school	3011 (93.1)	225 (6.9)	1.00 Ref	1.00
Did not complete secondary school	722 (88.5)	94 (11.5)	**1.74 (1.4-2.2)**	1.32 (0.9-1.7)

**Health care card**				
No	2990 (93.7)	202 (6.3)	1.00 Ref	1.00 Ref
Yes	743 (86.4)	117 (13.6)	**2.33 (1.8-2.9)**	1.44 (0.9-1.9)

**Parity**				
Multiparous	2066 (91.6)	189 (8.4)	1.00 Ref	1.00 Ref
Primiparous	1667 (92.8)	130 (7.2)	0.85 (0.7-1.1)	0.77 (0.6-1.1)

Multivariable logistic regression models were developed to provide a more precise estimate of associations between depressive symptoms, anxiety symptoms and co-morbid symptoms (primary outcomes) and the variable measuring stressful life events and social health issues (exposure of main interest). Other variables included in modelling analyses were: maternal age, relationship status, maternal country of birth, secondary education, parity and health care card. The latter was included as a measure of economic resources in preference to household income as there was less missing data for this item. Women with missing responses on any variable (or women who did not disclose their income by selecting response options 'not sure' of 'prefer not to answer') were excluded from these analyses. This left a total of 4063 cases for depression, 4078 cases for anxiety and 4052 cases for the co-morbid depression and anxiety analysis.

After adjusting for socio-demographic characteristics and parity, the significant associations between stressful life events and social health issues and the three outcome variables (anxiety symptoms, depressive symptoms, co-morbid anxiety and depressive symptoms) remained largely unchanged (Table 4 & 5). In each case the adjusted odds of psychological morbidity were raised five-fold for women experiencing three or more stressful life events or social health issues, with smaller significant effects for women reporting one to two events or issues. After adjusting for covariates, women born overseas in countries where English is not the primary language were significantly more likely to experience all forms of psychological morbidity; women with a healthcare card were significantly more likely to score as anxious; and women having their first baby less likely to score as depressed.

## Discussion

This study is the first to assess depression, anxiety and co-morbid anxiety and depression in a large population-based sample. Results show a concerning level of psychological morbidity with 17% experiencing depression and 13% experiencing anxiety at six months postpartum. Of particular concern is the identified level of co-morbidity with nearly one in ten women reporting both anxiety *and *depressive symptoms.

The high level of maternal anxiety and co-morbid anxiety and depression is generally not recognised in the perinatal depression literature [8,25]. Several exceptions to this come from Australia, including a small study using the DASS 21 which identified that 7% of women had symptoms of anxiety *and *depression at six weeks to six months postpartum [26]. A recent cohort study using the Edinburgh Postnatal Depression Scale (EPDS) [27] in combination with an interval symptom question and clinical interview identified that 8% had primary anxiety and 12% mixed depression and anxiety in the 6-8 month postnatal period [9].

The literature on life events draws attention to the significant impact of events such as moving house, the death of a family member and starting a new job. All of the women in this study were in the midst of managing one major life event: the birth of a baby and first few months after the birth. Many also experienced a range of stressful life events, such as the serious illness or death of a family member or friend, moving house or having a major illness themselves. In addition, some women experienced social health issues including serious family conflict, not having enough money to buy food, having a lot of bills they couldn't pay, or trouble associated with illicit drugs, alcohol or gambling. Data from 19 states participating in the USA PRAMS echoes these findings with 41% of women reporting at least one of several hardships (i.e. many bills that couldn't be paid, loss of job, separation or divorce) [1].

This study provides the first Australian population-based data on stressful life events and social health issues experienced by women in the six months following birth, and identifies a significant association with psychological morbidity. Australian and international studies [3,5,28,29] have also identified similar association between stressful life events and psychological ill-health, however are confined to perinatal depression as the outcome of interest. While it is not possible to determine causal pathways in an observational study such as this population-based survey, it is unlikely that such large effect sizes are explained by unmeasured confounders. The findings draw attention to social circumstances in women's lives that may place them at higher risk of poor mental health outcomes.

A number of studies have identified socio-demographic associations with postpartum depression, including factors associated with financial hardship [29], not being married [30] and migration of the mother from a country where English is not the primary community language [14,29]. At a univariate level this study supports these associations however only the maternal country of birth (non-English speaking background) remained statistically significant at the multivariate level. For the first time in a population-based sample we have identified that maternal country of birth is also associated with postpartum anxiety and co-morbid symptoms of depression and anxiety. In this study women with three or more stressful life events or social health issues were four to five times more likely to score above the cut-off scores on the DASS 21 for anxiety and depression, and almost six times more likely to have scores indicating co-morbid symptoms than women who did not report any stressful life events or social health issues in the months following birth. The strong relationship with postpartum emotional health is striking and illustrates the extent to which mental health in the postnatal period is related to exogenous factors over and beyond the immediate stresses associated with recovery after birth, and the physical and emotional demands of parenting.

The findings should alert health professionals engaged with women in the perinatal period to be attuned and responsive to potential stressors in women's lives. Providing perinatal care that is responsive to complexity in women's lives is challenging, especially in health systems characterised by short consultation times, limited integration between primary and specialist services, inadequate professional training in psychosocial assessment and treatment strategies, and limited availability of accessible and acceptable services for referral. The recently published NICE Guideline: *Pregnancy and complex social factors A model for service provision for pregnant women with complex social factors *provides a starting point for rethinking systems of care in ways that are likely to be more responsive to women experiencing major life events and social health issues in the months after childbirth [31]. Our findings suggest that the effectiveness of treatment options for depression and anxiety may be limited if the potential impact of stressful life events and social health issues is not recognised. Cognitive Behavioural Therapy and pharmacological approaches have proven effectiveness in treatment of depression, but may be less effective in resolving maternal depression and anxiety if social determinants are not addressed.

The focus of this paper has been on the postnatal period, but women are likely to experience a similar range and number of stressors during pregnancy [11] with evidence that life stressors in pregnancy are associated with depression after birth [2,29]. We hope our findings invigorate debate about the role of antenatal care in acting early to identify and support women at risk of poor mental health outcomes; about whose responsibility it is to provide support to women experiencing social adversity during and after pregnancy; and how services can work together to provide care that addresses the social context of women's lives and impact of a range of common life events and social health issues. This will require change across a currently fragmented system of perinatal care with integration between interventions to counter the provision of distinct vertical programs [32]. Reform strategies will need to be multi-faceted; focus on a continuum of care spanning maternal, newborn and child health; and strongly grounded in an integrated approach to service delivery that addresses underlying social determinants [33].

Strengths of this study include: a population-based sample with adequate power to detect differences between sub-groups including groups of women who are less likely to participate in surveys of this kind, for example younger women; the capacity to identify the extent of selection bias by comparing study participants with women giving birth in the study period using routine data collections in both states; a largely representative sample in terms of important obstetric covariates (e.g. method of birth, parity); and use of standardised measures for anxiety and depressive symptoms. The DASS-21 was used in preference to the EPDS used in previous Victorian surveys as it includes a scale for anxiety symptoms, and because the EPDS is now in increasingly common use in clinical settings in Victoria and South Australia, which compromises its value as a research instrument. The DASS-21 has good psychometric properties and the depression scale correlates well with the EPDS. The 21 item DASS was well completed, with 2% of women unable to be categorised due to missing values. Prevalence of depressive symptoms was similar to estimates of prevalence found in other studies including the three previous Victorian surveys using the EPDS [2,18,34,35].

The response fraction was similar to other recent population-based surveys [e.g. [36]]. Compared to the Victorian surveys of recent mothers of 1998, 2004 and 2000 [10-12] this study was larger and several processes differed in order to conform to ethics and privacy requirements. We are aware that several participating hospitals found managing some of the processes associated with identification of births, exclusions and mail-out procedures challenging. The quoted response fraction is conservative, and it is likely that the response from women who actually received a mailed survey was higher.

Like other postal surveys of this kind the survey under-represents women who are single, young, born overseas in a non-English speaking background country and not privately insured at the time of birth [10,11]. These factors limit the generalisbility of the findings. It is likely that prevalence estimates for depression and anxiety underestimate the true prevalence due to the under representation of younger women and women of non-English speaking background in our sample.

## Conclusion

Given the high prevalence of psychological morbidity and the association with social health issues experienced by women in the months following birth, it is critical that health professionals providing care to women in the perinatal period be alert to the range of social health issues. Awareness and recognition needs to be followed through with care which it is sensitive and supportive of women's individual health and social circumstances. Integrated care pathways would enable care providers to respond with a coordinated approach to on-going support and management of women at risk of poor mental health outcomes. Care that is responsive to women's social health may well lead to improvements in women's emotional wellbeing following birth.

## Competing interests

The authors declare that they have no competing interests.

## Authors' contributions

The study was conceived and designed by SB and JY; JY and GS participated in coding of questionnaires and undertook the survey data analysis. All authors contributed to the manuscript and read and approved the final version.

## Pre-publication history

The pre-publication history for this paper can be accessed here:

http://www.biomedcentral.com/1471-2458/10/771/prepub

## References

[B1] Braveman P Marchi K Kim S Metzler M Stancil T Libet M Poverty, near poverty, and hardship around the time of pregnancy Matern Child Health J 2010 14 20 35 10.1007/s10995-008-0427-0 19037715

[B2] O'Hara M Swain A Rates and risk of postpartum depression - a meta-analysis Int Rev Psychiatry 1996 8 37 54

[B3] Rubertsson S Wickberg B Gustavsson P Raedestad I Depressive symptoms in early pregnancy, two months and one year postpartum-prevalence and psychosocial risk factors in a national Swedish sample Arch Womens Ment Health 2005 8 97 104 10.1007/s00737-005-0078-8 15883652

[B4] Johnstone S Boyce P Hickey A Morris-Yates A Harris M Obstetric risk factors for postnatal depression in urban and community samples Aust N Z J Psychiatry 2001 35 69 74 10.1046/j.1440-1614.2001.00862.x 11270460

[B5] Herrick H The effect of stressful life events on postpartum depression: Results from the 1997-1998 North Carolina Pregnancy Risk Assessment Monitoring System (PRAMS) SCHS Stud 2000 121 1 9

[B6] Wisner L Chambers C Sit D Postpartum depression: a major public health problem JAMA 2006 296 2616 2618 10.1001/jama.296.21.2616 17148727

[B7] Antenatal and postnatal mental health. The NICE Guideline on clinical management and service guidance 2007 http://www.nice.org.uk/nicemedia/live/11004/30431/30431.pdf Accessed 21 November 2010 21678630

[B8] Mathey S Barnett B Howie P Kavanagh K Diagnosing postpartum depression in mothers and fathers: Whatever happened to anxiety? J Affect Disord 2003 74 139 147 10.1016/S0165-0327(02)00012-5 12706515

[B9] Austin M-P Priest S Reilly N Wilhelm K Saint K Parker G Depressive and anxiety disorders in the postpartum period: how prevalent are they and can we improve their detection? Arch Womens Ment Health 2010 13 395 401 10.1007/s00737-010-0153-7 20232218

[B10] Masi G Millepiedi S Mucci M Poli P Bertini N Milantoni L Generalized anxiety disorder in referred children and adolescents J Am Acad Child Adolesc Psychiatry 2004 43 752 760 10.1097/01.chi.0000121065.29744.d3 15167092

[B11] Dole N Savitz DA Hertz-Picciotto I Siega-Riz AM McMahon MJ Buekens P Maternal stress and preterm birth Am J Epidemiol 2003 157 14 24 10.1093/aje/kwf176 12505886

[B12] Glasheen C Richardson G Fabio A A systematic review of the effects of postnatal maternal anxiety on children Arch Womens Ment Health 2010 13 61 74 10.1007/s00737-009-0109-y 19789953 PMC3100191

[B13] Benoit C Westfall R Treloar A Phillips R Jansson M Social factors linked to postpartum depression: A mixed-methods longitudinal study J Mental Health 2007 16 719 730 10.1080/09638230701506846

[B14] Astbury J Brown S Lumley J Small R Birth events, birth experiences and social differences in postnatal depression Aust J Public Health 1994 18 176 184 10.1111/j.1753-6405.1994.tb00222.x 7948335

[B15] Gross K Wells C Radigan-Garcia A Dietz P Correlates of self-reports of being very depressed in the months after delivery: results form the Pregnancy Risk Assessment Monitoring System Mat Child Health J 2002 6 247 253 10.1023/A:1021110100339 12512766

[B16] Matthey S Phillips J White T Glossop P Hopper U Panasetis P Petridis A Larkin M Barnett B Routine psychosocial assessment of women in the antenatal period: frequency of risk factors and implications for clinical services Arch Womens Ment Health 2004 7 223 229 10.1007/s00737-004-0064-6 15338316

[B17] Australian Bureau of Statistics, Household income and income distribution 2007-2008. Canberra 2009

[B18] Brown S Lumley J The 1993 Survey of Recent Mothers: issues in design, analysis and influencing policy Int J Qual Health Care 1997 9 265 277 9304425 10.1093/intqhc/9.4.265

[B19] Brown S Bruinsma F Darcy M-A Small R Lumley J Early discharge: no evidence of adverse outcomes in three consecutive population-based Australian surveys of recent mothers conducted in 1989, 1994 and 2000 Paediatric and Perinatal Epidemiology 2004 18 202 213 10.1111/j.1365-3016.2004.00558.x 15130160

[B20] Brown S Lumley J Satisfaction with care in labor and birth: a survey of 790 Australian women Birth 1994 21 4 13 10.1111/j.1523-536X.1994.tb00909.x 8155224

[B21] Lovibond SH Lovibond PF Manual for the Depression Anxiety Stress Scales 1995 2 Sydney: Psychology Foundation

[B22] Lovibond PF Lovibond SH The structure of negative emotional states: comparison of the Depression Anxiety Stress Scales (DASS) with the Beck Depression and Anxiety Inventories Behaviour Research and Therapy 1995 33 335 343 10.1016/0005-7967(94)00075-U 7726811

[B23] Colley Gilbert BJ Shulman HB Fischer LA Rogers MM The Pregnancy Risk Assessment Monitoring System (PRAMS): methods and 1996 response rates from 11 states Matern Child Health J 1999 3 199 209 10.1023/A:1022325421844 10791360

[B24] Stata Corporation Stata Statistical Software Release 11 2009 College Station, Texas: Stat Corporation

[B25] Fisher J Feekrey C Rowe-Murray H Nature, severity and correlates of psychological distress in women admitted to a private mother-baby unit J Paediatr Child Health 2002 38 140 145 10.1046/j.1440-1754.2002.00723.x 12030994

[B26] Miller RM Pallant JF Negri LM Anxiety and stress in the postpartum: Is there more to postnatal distress than depression? BMC Psychiatry 2006 6 12 10.1186/1471-244X-6-12 16563155 PMC1450275

[B27] Cox J Holden J Sagovski R Detection of postnatal depression: development of the 10-item Edinburgh Postnatal depression Scale Br J Psychiatry 1987 150 782 786 10.1192/bjp.150.6.782 3651732

[B28] Edwards B Galletly C Semmler-Booth Dekker G Does antenatal screening for psychosocial risk factors predict postnatal depression? A follow-up study of 154 women in Adelaide, South Australia A N Z J Psychiatry 2008 42 51 55 10.1080/00048670701739629 18058444

[B29] Dennis C-L Janssen P Singer J Identifying women at-risk for postpartum depression in the immediate postpartum period Acta Psychiatr Scan 2004 110 338 346 10.1111/j.1600-0447.2004.00337.x 15458557

[B30] Beck C Predictors of postpartum depression: an update Nurs Res 2001 50 275 285 10.1097/00006199-200109000-00004 11570712

[B31] NICE clinical guideline 110 - Pregnancy and complex social factors: a model for service provision for pregnant women with complex social factors 2010 http://www.nice.org.uk/guidance Accessed 24 September 2010 21977548

[B32] Fenk J Reinventing primary health care: the need for systems integration Lancet 2009 374 173 10.1016/S0140-6736(09)60693-0 19439350

[B33] Ekman B Pathmanathan I Liljestrand J Integrating health interventions for women, newborn babies and children; a framework for action Lancet 2008 372 990 1000 10.1016/S0140-6736(08)61408-7 18790321

[B34] Mann R Gilbody S Adamson J Prevalence and incidence of postnatal depression: what can systematic reviews tell us? Arch Womens Ment Health 2010 13 295 305 10.1007/s00737-010-0162-6 20440525

[B35] Brown S Lumley J Physical health problems after childbirth and maternal depression at six to seven months postpartum BJOG 2000 107 1194 1201 10.1111/j.1471-0528.2000.tb11607.x 11028568

[B36] Maconochie N Doyle P Prior S The National Women's Health Study: assembly and description of a population-based reproductive cohort BMC Public Health 2004 4 35 10.1186/1471-2458-4-35 15298712 PMC514555

